# FOXM1 Maintains Homeostasis and Self-Renewal in Wharton’s Jelly Mesenchymal Stem Cells

**DOI:** 10.3390/genes16121517

**Published:** 2025-12-18

**Authors:** Nan Li, Qiang Wu

**Affiliations:** 1 Faculty of Chinese Medicine and the State Key Laboratory of Mechanism and Quality of Chinese Medicine, Macau University of Science and Technology, Macau 999078, China; 2 The State Key Laboratory of Mechanism and Quality of Chinese Medicine, Macau University of Science and Technology, Macau 999078, China

**Keywords:** FOXM1, WJ-MSCs, homeostasis, stem cell identity

## Abstract

Background: The transcription factor FOXM1 is a master regulator of the cell cycle and is implicated in various cell fate decisions. However, its functional role and regulatory network in human Wharton’s jelly mesenchymal stem cells (WJ-MSCs) remain poorly defined. This study aimed to elucidate the comprehensive function of FOXM1 in maintaining WJ-MSC stemness, proliferation, and survival, and to delineate the underlying molecular mechanisms. Methods: We used RNA Interference to knock down FOXM1 in WJ-MSCs. The phenotypic impacts were assessed through CCK-8, colony formation, migration, and flow cytometry assays. We analyzed transcriptomic changes using RNA-seq and verified the results through qRT-PCR and Western blotting. Results: Knockdown of FOXM1 significantly reduced the expression of core pluripotency factors (OCT4, SOX2, and NANOG), impairing stem cell identity and abolishing colony formation and migration capacities. Furthermore, FOXM1 deficiency induced G0/G1 phase cell cycle arrest, downregulated CCND1, and triggered apoptosis through a mechanism involving p53 accumulation, an increased BAX/BCL-2 ratio, and Caspase-3 activation. RNA-seq analysis further corroborated the systematic downregulation of cell cycle pathways and upregulation of apoptotic pathways upon FOXM1 deficiency. Conclusions: Our findings establish FOXM1 as a critical regulatory node that integrates stem cell identity with proliferative and survival signals to maintain WJ-MSC homeostasis. This study redefines FOXM1’s role in stem cell biology and provides a theoretical foundation for enhancing the therapeutic efficacy of WJ-MSCs by modulating this key factor.

## 1. Introduction

Increasing evidence suggests that an imbalance in the pluripotency of stem cells not only promotes spontaneous differentiation but also compromises their therapeutic efficacy [1]. Therefore, effectively maintaining the undifferentiated state and therapeutic potential of mesenchymal stem cells (MSCs) during in vitro expansion is a critical challenge for their clinical translation [2]. Wharton’s jelly MSCs (WJ-MSCs) represent a highly promising cell source for overcoming this challenge. They possess the classic MSC features of unlimited self-renewal capacity, multidirectional differentiation potential, and immunoregulatory abilities [3]. WJ-MSCs also serve as an important source of primitive cells that originate from the ectoderm of umbilical cord tissue and are classified as medical waste discarded after delivery [4]. They are easily accessible and raise no ethical concerns, and no tumorigenicity has been observed. Importantly, WJ-MSCs can consistently and stably express pluripotency core markers such as OCT4, SOX2, and NANOG at high levels [5]. Even after multiple passages, WJ-MSCs can maintain their stemness and are less prone to aging [6]. These properties make them exceptionally suitable for clinical therapies and large-scale production [7].

The therapeutic potential of MSCs primarily depends on their ability to maintain an undifferentiated state and multidirectional differentiation capacity, demonstrating great promise in the fields of regenerative medicine and tissue engineering [8,9]. WJ-MSCs, in particular, have diverse clinical applications. For example, they can be induced to differentiate into neural-like cells for the treatment of neurological disorders [10] or into hepatocyte-like cells as a potential cell source for liver transplantation [11]. Additionally, their ability to differentiate into osteoblasts shows promise in the intervention of osteoporosis [12], and various other applications [13,14,15]. This ability to maintain an undifferentiated state during rapid proliferation suggests the presence of a complex molecular regulatory network within the cells.

Recent research has uncovered a direct molecular link between cell cycle regulation mechanisms and the maintenance of stem cell pluripotency [16]. For instance, c-Myc, a core cell cycle regulator, not only promotes the transition from G1 to S phase but also plays a critical role in maintaining the stability of the self-renewal and pluripotency gene networks [17]. This mechanism elucidates a key characteristic of stem cells: the ability to sustain an undifferentiated state while actively proliferating [18]. Consequently, we speculate that proliferative factors and differentiation inhibitory factors may form a synergistic regulatory network in stem cells. Based on this, we hypothesize that a center regulatory network can concurrently manage both “proliferative vigor” and “cell identity maintenance,” hence functioning as a “stemness guardian”. Therefore, exploring the molecular basis for maintaining the identity of WJ-MSCs becomes particularly important.

FOXM1, as a member of the FOX transcription factor family, plays a central role in regulating cell proliferation [19]. It governs the transitions between G1/S and G2/M phases, orchestrates DNA replication processes, and is pivotal in mitotic progression [20,21]. Indeed, multiple studies have confirmed that FOXM1 is a primary driver of cell proliferation across various tissues [22,23]. It achieves this function by regulating the expression of cell cycle-related genes, such as Cyclin B1 and PLK1 [24]. It is noteworthy that FOXM1 is highly expressed in various stem cells and is a key element in maintaining cellular proliferation homeostasis [25]. However, the expression characteristics, biological functions, and specific regulatory mechanisms of FOXM1 in more primitive WJ-MSCs remain to be elucidated.

Increasing evidence suggests that the function of FOXM1 is significantly context-dependent. Its role can vary based on the tumor microenvironment and other external factors. In certain cases, such as in lung cancer [26], breast cancer [27], and colorectal cancer [28], abnormal high expression of FOXM1 promotes oncogenic processes such as cell proliferation, invasion, and metastasis. This overexpression is associated with poor clinical outcomes for patients, highlighting FOXM1’s role as a potential therapeutic target in cancer treatment [29]. However, in other cases, such as promoting skin wound healing in diabetic foot ulcers by regulating neutrophil extracellular traps, FOXM1 exhibits a protective role in tissue regeneration. This indicates that FOXM1 may have different functions in various biological contexts, contributing to both tumor progression and tissue repair and regeneration [30]. During embryonic development, FOXM1 is also essential. Mice with a knockout of the *Foxm1* gene experience postnatal death due to lung developmental defects [31]. At the cellular level, downregulation of FOXM1 leads to cell cycle arrest, decreased proliferation capacity, and increased genomic instability [32].

The therapeutic potential of WJ-MSCs is critically dependent on their proliferative capacity during in vitro expansion. However, the role of FOXM1 in integrating cell cycle and apoptotic pathways to maintain WJ-MSC homeostasis has remained unclear. This study aimed to systematically investigate FOXM1’s functions and test the hypothesis that it acts as a “central regulator” that coordinates proliferation and apoptosis. Our work is the first to define this core regulatory function of FOXM1, thereby providing a theoretical foundation and potential intervention targets for optimizing in vitro expansion and enhancing the efficacy of WJ-MSC-based therapies.

## 2. Materials and Methods

### 2.1. Cell Source and Culture

Human Wharton’s jelly mesenchymal stem cells (WJ-MSCs) obtained from ATCC (Manassas, VA, USA; cat. PCS-500-010™, neonate donor). WJ-MSCs were cultured in OriCell^®^ Human Umbilical Cord Mesenchymal Stem Cell Complete Medium (HUXUC-90011, Cyagen Biosciences, Suzhou, China) supplemented with 12% OriCell^®^ Fetal Bovine Serum and Culture Supplement (Cyagen Biosciences, Suzhou, China). WJ-MSCs were seeded into 6-well plates (Corning, Glendale, AZ, USA) at a density of 1 × 10^5^ cells per well. All cultures were incubated in a humidified atmosphere at 37 °C with 5% CO_2_ (Thermo Fisher Scientific, Waltham, MA, USA). To ensure optimal nutrient supply, the culture medium was refreshed every 24 h. Upon reaching 80–90% confluence, typically within 48 to 72 h, the cells were subcultured to sustain their logarithmic growth and viability. All experiments in this study were carried out using cells between the 3rd and 6th passages.

### 2.2. RNA Interference (siRNA)

WJ-MSCs were plated into 6-well culture plates at a density of 2 × 10^5^ cells per well. Two experimental groups were established: one group received *FOXM1*-siRNA (cat. sc-43769; Santa Cruz Biotechnology, Dallas, TX, USA) targeting all major isoforms (pan-isoform siRNA), while the other group received control-siRNA (cat.sc-37007; Santa Cruz Biotechnology). To perform transfection, siRNA-LipoRNAi™ complexes (Invitrogen, Dallas, TX, USA) were prepared in serum-free Opti-MEM™ medium (Gibco, Dallas, TX, USA). These complexes were then added to the wells containing the pre-plated cells, following the manufacturer’s instructions. After the transfection process, the cells were cultured in high-serum medium, specifically DMEM supplemented with 12% FBS (Gibco). The cells were incubated under standard conditions of 37 °C and 5% CO_2_ for a duration of 48 h.

### 2.3. Real-Time Quantitative PCR (RT-qPCR)

Total RNA from the cells was extracted using TRIzol Reagent (Thermo Fisher Scientific) according to the manufacturer’s instructions. The cDNA was synthesized from the extracted RNA using the NovoScript^®^ III Reverse Transcriptase kit (Invitrogen). For quantitative real-time PCR analysis, Fast SYBR Green Master Mix (Bio-Rad Laboratories, Hercules, CA, USA) was utilized. The PCR procedure began with an initial denaturation step at 95 °C for 10 min. This was followed by 40 cycles, each consisting of denaturation at 95 °C for 15 s, annealing at 60 °C for 1 min, and extension at 72 °C for 1 min. To determine mRNA levels, the relative quantification was performed using the 2^−ΔΔCt^ method, with *GAPDH* as the internal control gene. Details of the primers used for RT-qPCR were provided in Table S1.

### 2.4. Western Blotting

For the Western blotting experiment, cells were first lysed on ice using RIPA lysis buffer (P0013C, Beyotime, Shanghai, China). The total protein concentration was then measured using a BCA assay kit (P0010S, Beyotime, Shanghai, China). Next, total proteins were separated using 10% SDS-PAGE (Bio-Rad) and transferred to a PVDF membrane (Millipore, Burlington, MA, USA) through wet transfer. After transferring, the membrane was blocked with 5% non-fat milk for 1 h to reduce non-specific binding. The membrane was then incubated with the primary antibody (listed in Table S2, including catalog numbers, clone identifiers and dilutions) overnight at 4 °C, followed by the corresponding HRP-conjugated secondary antibodies (in Table S2) for 2 h at room temperature. Finally, the membrane was washed with TBST, and the signal was developed using ECL reagent (Pierce, Thermo™ Fisher, Dallas, TX, USA). The imaging system (Bio-Rad) was used to capture the signals. The list of all primary and secondary antibodies used is provided in Table S2.

### 2.5. RNA Sequencing

Prior to RNA extraction, WJ-MSCs underwent *FOXM1* knockdown treatment. Total RNA was extracted when the cultures reached approximately 80% confluence and displayed healthy morphology. Sequencing libraries were constructed from qualified RNA using the VAHTS Universal V10 RNA-seq Library Prep Kit for Illumina (premixed, Vazyme, cat. no. NR616-02, San Diego, CA, USA). The prepared libraries were sequenced on the Illumina NovaSeq X Plus platform, generating 150 bp paired-end reads. For data analysis, the raw sequencing data underwent quality control using Fastp. High-quality readings were then aligned to the human reference genome for differential expression analysis.

### 2.6. Colony Formation

A colony formation assay was performed to assess the clonogenic potential of WJ-MSCs following *FOXM1* knockdown. Cells were transfected with either a negative control siRNA or *FOXM1* siRNA. After transfection, the cells were harvested and plated into 6-well culture plates (Corning, Glendale, AZ, USA) at a density of 1 × 10^3^ cells per well. The cultures were maintained with routine medium replacement every 3 to 4 days. Upon completion of a 14-day incubation period, the resulting cell colonies were subjected to staining. Briefly, the culture medium was aspirated, and the cells were fixed with formaldehyde. The fixed colonies were then stained with 0.2% crystal violet (1 mL per well) for 30 min at ambient temperature. Subsequently, unbound dye was thoroughly removed by gently washing the plates three times with phosphate-buffered saline (PBS). Finally, the plates were air-dried in a ventilated space, and the stained colonies were documented by photography for morphological analysis.

### 2.7. Wound Healing Assay

WJ-MSCs from the control and experimental groups were seeded in 6-well plates and cultured for 24 h until the cell density exceeded 80%. A scratch was then made on the cell layer using a 200 µL pipette tip. The plates were then washed with PBS to remove any floating cells, and the culture medium was replaced with serum-free medium. The cells were observed under an Olympus microscope (Olympus, Tokyo, Japan) to capture images of the scratched area. Duplicate wells from each control and experimental group were analyzed. The scratch distance was quantified and compared to the difference from the initial scratch distance. The percentage represents the degree of scratch closure, i.e., the migration rate. Statistical analysis was performed to assess the significance of the differences between the two groups.

### 2.8. CCK-8 Cell Proliferation Assay

WJ-MSCs were seeded in 96-well plates (Corning, USA) at a density of 5 × 10^3^ cells/well. Cells were transfected with *FOXM1* siRNA and control siRNA at 24, 48, and 72 h. Before assessing cell proliferation at each transfection time point, the following steps were performed: the culture medium was removed, and 100 μL of complete DMEM (containing 10 μL of CCK-8 reagent, Beyotime Biotechnology) was added to each well. The plates were then incubated at 37 °C in a 5% CO_2_ incubator for 1 h. After incubation, the optical density (OD) at 450 nm was measured using an automated microplate reader (Hitachi, Tokyo, Japan). Data were recorded at 24, 48, and 72 h post-transfection.

### 2.9. Cell Cycle and Apoptotic Analysis

For cell cycle analysis, WJ-MSCs were seeded in culture dishes and transfected with either negative control or *FOXM1*-siRNA for 48 h. After transfection, the cells were collected and resuspended in pre-chilled PBS buffer. The cells were then mixed with 70% ethanol that had been cooled to 4 °C and fixed at this temperature for 24 h. Staining was performed using the Cell Cycle Staining Kit (100-107, GOONIE, Guangzhou, China), following the manufacturer’s instructions closely. After a 30 min incubation at 37 °C in the dark, the samples were analyzed using a FACScan flow cytometer (Becton Dickinson, Franklin Lakes, NJ, USA) to determine cell cycle distribution.

For apoptosis detection, the cells were similarly transfected with negative control or *FOXM1*-siRNA for 48 h. According to the manufacturer’s guidelines, the cells were collected and stained using the Annexin V-FITC/PI Apoptosis Kit (100-101, GOONIE, Guangzhou, China). Approximately 1 × 10^5^ cells per sample were resuspended in 100 μL of binding buffer. The cells were then stained with 5 μL of Annexin V-FITC and 5 μL of Propidium Iodide (PI) for 15 min at room temperature in the dark. After a 30 min incubation, the samples were analyzed for apoptosis using the FACScan flow cytometer (Becton Dickinson). Fluorescence compensation was performed using single-stained controls (cells stained with Annexin V-FITC only or PI only) to correct for spectral overlap. All flow cytometry results were analyzed with FlowJo software v10 (Becton Dickinson, Franklin Lakes, NJ, USA).

### 2.10. Statistical Analysis

Statistical analysis of the data was carried out using GraphPad Prism 9.5, with significance determined through Student’s *t*-test. The experiments were conducted in triplicate to ensure reliability, and results were expressed as mean ± standard deviation from at least three independent experiments. A probability value of less than 0.05 was considered statistically significant, with the following thresholds applied: * *p* < 0.05, ** *p* < 0.01, *** *p* < 0.001.

## 3. Results

### 3.1. FOXM1 Depletion Compromises Clonogenicity and Survival of WJ-MSCs

**Figure 1:**
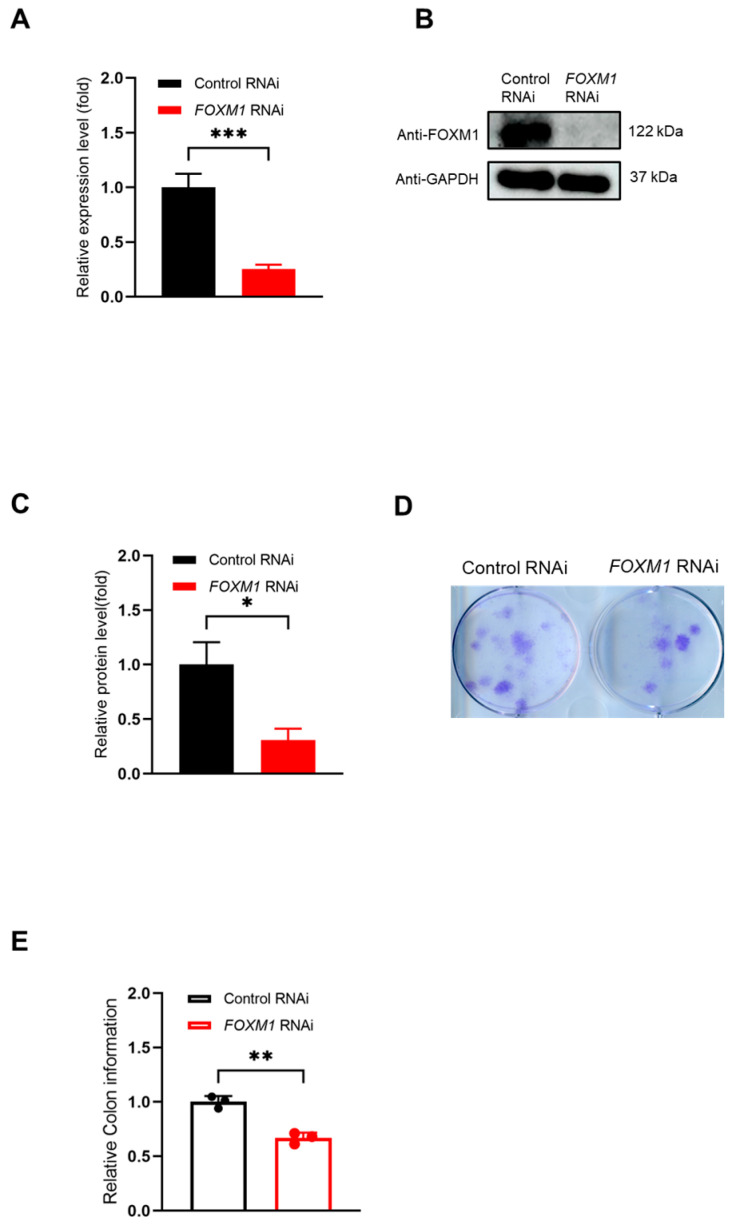
FOXM1 depletion affects the clonogenicity of WJ-MSCs. (**A**) *FOXM1* gene expression in WJ-MSCs was assessed by RT-qPCR. (**B**) FOXM1 protein expression levels were assessed by Western blot. (**C**) Protein quantification of FOXM1. (**D**) Clony formation assay using crystal violet staining. (**E**) *FOXM1* RNAi significantly reduced the area of colony coverage. * *p* < 0.05, ** *p* < 0.01, and *** *p* < 0.001.

To investigate the role of FOXM1 in WJ-MSCs, we first achieved efficient knockdown by transfecting cells with *FOXM1*-targeting siRNA, using a non-targeting siRNA as a negative control. The success of the knockdown was confirmed at both the transcriptional and protein levels. RT-qPCR analysis showed that *FOXM1* mRNA expression was reduced to less than 20% of the level in the control group (Figure 1A). Consistent with this, Western blot analysis demonstrated a significant decrease in FOXM1 protein expression (Figure 1B,C).

Having established the model, we assessed the functional impact of FOXM1 loss on proliferative capacity using a colony formation assay. The control group formed a high number of colonies, whereas the *FOXM1*-knockdown group exhibited a stark reduction in both the number and coverage area of colonies (Figure 1D,E, ** *p* < 0.01). These results demonstrate that FOXM1 is essential for the clonogenic proliferation and survival of WJ-MSCs.

### 3.2. Global Gene Expression Changes upon FOXM1 Knockdown

**Figure 2:**
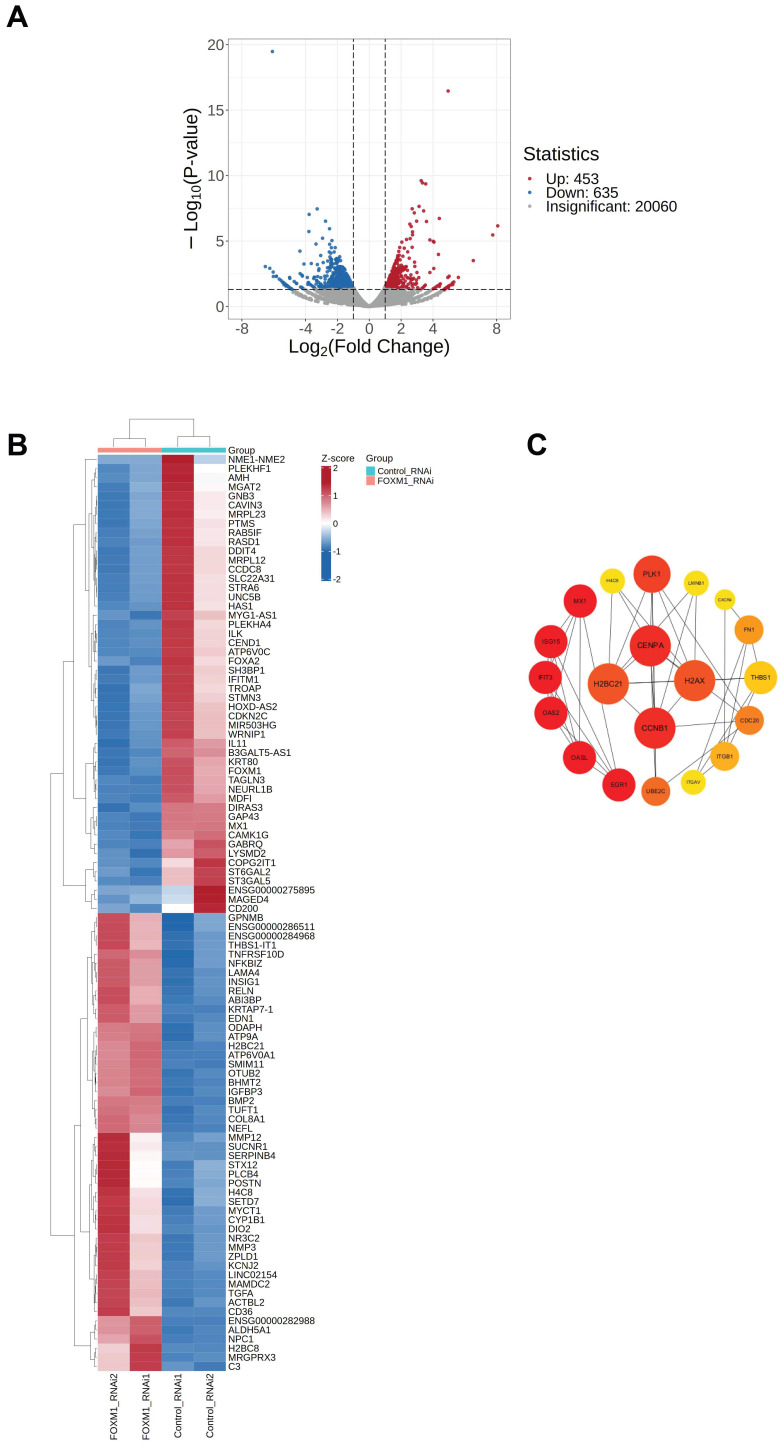
Analysis of RNA-seq results following *FOXM1* knockdown. (**A**) Visualization of RNA-seq results with Volcano plot. (**B**) Visualization of differentially expressed genes with Heatmap (red color for up-regulation and blue color for down-regulation). (**C**) A deeper red color of the Hub gene indicates a higher score.

To elucidate the role of the transcription factor FOXM1 in WJ-MSC pluripotency, we performed RNA-seq analysis following *FOXM1* knockdown. Using a threshold of |log2FoldChange| ≥ 1 and a *p*-value < 0.05, we identified 453 significantly upregulated and 635 downregulated genes (Figure 2A). Unsupervised clustering of the top 100 most significantly altered genes (50 up, 50 down) revealed a clear distinction between FOXM1-depleted and control cells (Figure 2B). Gene ontology analysis indicated that these differentially expressed genes were primarily enriched in processes including cell cycle regulation, signal transduction, and immune responses.

To identify key network nodes, we constructed a protein–protein interaction network using Cytoscape v3.10.1. The cytoHubba plugin highlighted the top 20 hub genes, which are strongly associated with the cell cycle, DNA damage repair, and immune signaling (Figure 2C). Notably, the cell cycle gene *CCNB1* was among the highest-ranked hub genes, underscoring a potential central role for FOXM1 in regulating cell proliferation in WJ-MSCs.

### 3.3. FOXM1 Knockdown Leads to Identity Disruption in WJ-MSCs

**Figure 3:**
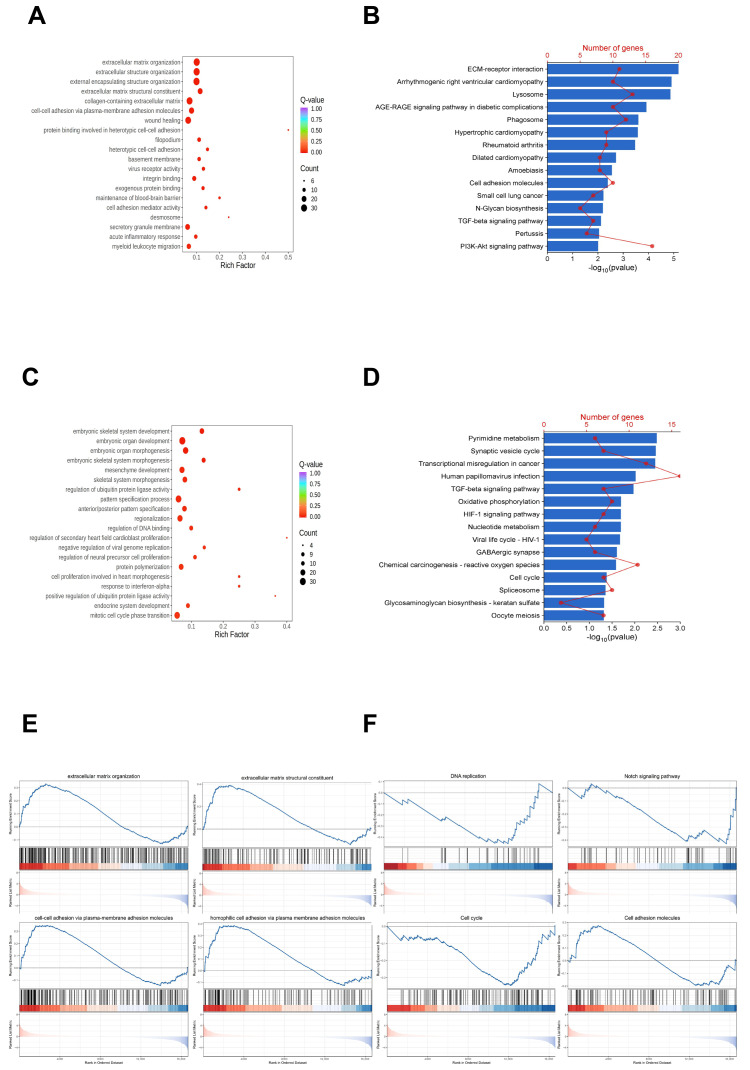
FOXM1-related pathways were identified by differential gene enrichment analysis. (**A**) GO enrichment for up-regulated genes. (**B**) The top 15 KEGG pathways enriched by up-regulated genes. (**C**) GO enrichment for down-regulated genes. (**D**) The top 15 KEGG pathways enriched by down-regulated genes. (**E**) GO analysis of GSEA. (**F**) KEGG analysis of GSEA.

To further explore how *FOXM1* knockdown affects biological functions of WJ-MSCs, we conducted Gene Ontology (GO) and KEGG analyses on both upregulated and downregulated genes. The GO analysis revealed that upregulated genes were significantly enriched in key biological processes such as extracellular matrix organization, cell adhesion, and wound healing. In contrast, the downregulated genes were primarily associated with biological processes related to embryonic development, cell proliferation and differentiation, and protein degradation and regulation (Figure 3A,C).

To gain more insights into the signaling pathways in *FOXM1*-knockdown WJ-MSCs, we performed KEGG analysis on the upregulated and downregulated genes. We selected the top 15 pathways with a *p*-value < 0.05 to indicate significant enrichment. The results showed that upregulated genes were mainly linked to pathways involving extracellular matrix remodeling, lysosomal function alterations, and mechanisms related to diabetes complications. Conversely, pathways associated with downregulated genes were primarily related to cellular metabolism, signal transduction, cell cycle regulation, and rheumatoid arthritis (Figure 3B,D). Additionally, Gene Set Enrichment Analysis (GSEA) indicated that the enrichment results were closely related to intercellular adhesion, extracellular matrix interactions, as well as signaling pathways involved in the cell cycle and apoptosis (Figure 3E,F).

To visually confirm the coordinated changes within these highlighted pathways and to provide a global view of gene expression, we generated expression heatmaps for all genes comprising the Cell Cycle (Figure S1A) and Apoptosis pathways (Figure S1B). We identified these two pathways as the most significantly enriched and phenotypically relevant pathways. The heatmaps clearly demonstrated a consistent downregulation trend across most genes in the cell cycle pathway and a concurrent upregulation trend in the apoptosis pathway. Together, these results demonstrated that FOXM1 may play multifaceted roles in WJ-MSCs.

### 3.4. The Important Regulatory Role of FOXM1 in WJ-MSCs

**Figure 4:**
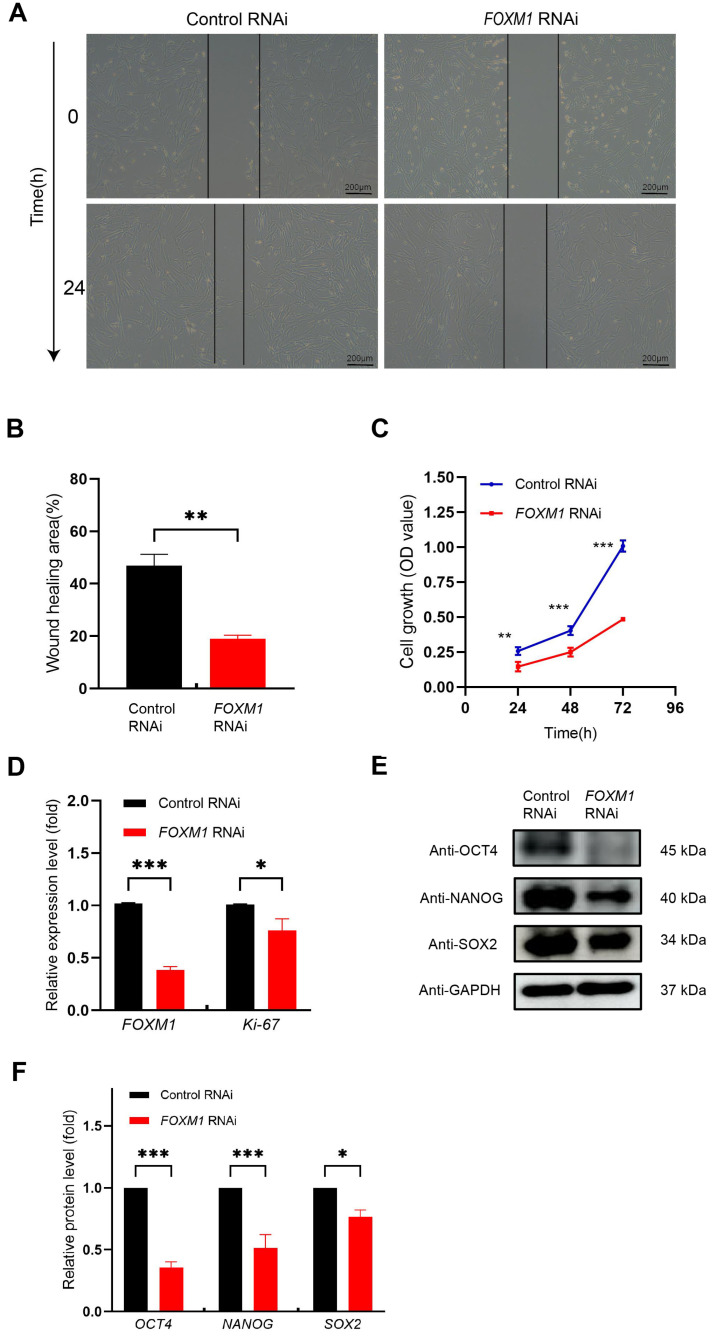
Knockdown of *FOXM1* inhibits WJ-MSC migration, proliferation, and pluripotency. (**A**) Wound healing of WJ-MSCs treated with control RNAi and *FOXM1* RNAi after scratch wounding. (**B**) Quantification of wound distance represented the results of three independent experiments. (**C**) Cell proliferation was assessed using the CCK8 assay. Each time point in each row was n = 5. (**D**) Ki-67 expression was measured using RT-qPCR. (**E**) Western blot analysis was used to assess pluripotency protein expression. (**F**) Quantitative analysis of pluripotency protein expression was performed. * *p* < 0.05, ** *p* < 0.01, and *** *p* < 0.001.

We next investigated the impact of *FOXM1* knockdown on the migration, proliferation, and pluripotency of WJ-MSCs. The migratory capacity of FOXM1-knockdown cells was significantly impaired, as demonstrated by a wound healing assay showing a markedly reduced migration rate after 24 h compared to the control group (Figure 4A,B).

Consistent with this functional deficit, *FOXM1* knockdown also severely compromised proliferative capacity. CCK-8 assays revealed a significant reduction in cell proliferation after 48 h (Figure 4C, *** *p* < 0.001). This was further supported by a concomitant decrease in the mRNA expression of both *FOXM1* itself and the proliferation marker *MKI67* (which encodes Ki-67) (Figure 4D, ** *p* < 0.001, * *p* < 0.05).

Furthermore, FOXM1 depletion critically impaired stem cell pluripotency. Western blot analysis showed a substantial reduction in the core pluripotency factors OCT4, NANOG, and SOX2 (Figure 4E). Densitometric quantification confirmed these decreases were statistically significant (Figure 4F). Collectively, these findings suggest that FOXM1 serves as an essential factor for maintaining the migratory, proliferative, and pluripotent properties of WJ-MSCs.

### 3.5. FOXM1 Knockdown Inhibits WJ-MSCs Proliferation by Regulating the Cell Cycle Process

**Figure 5:**
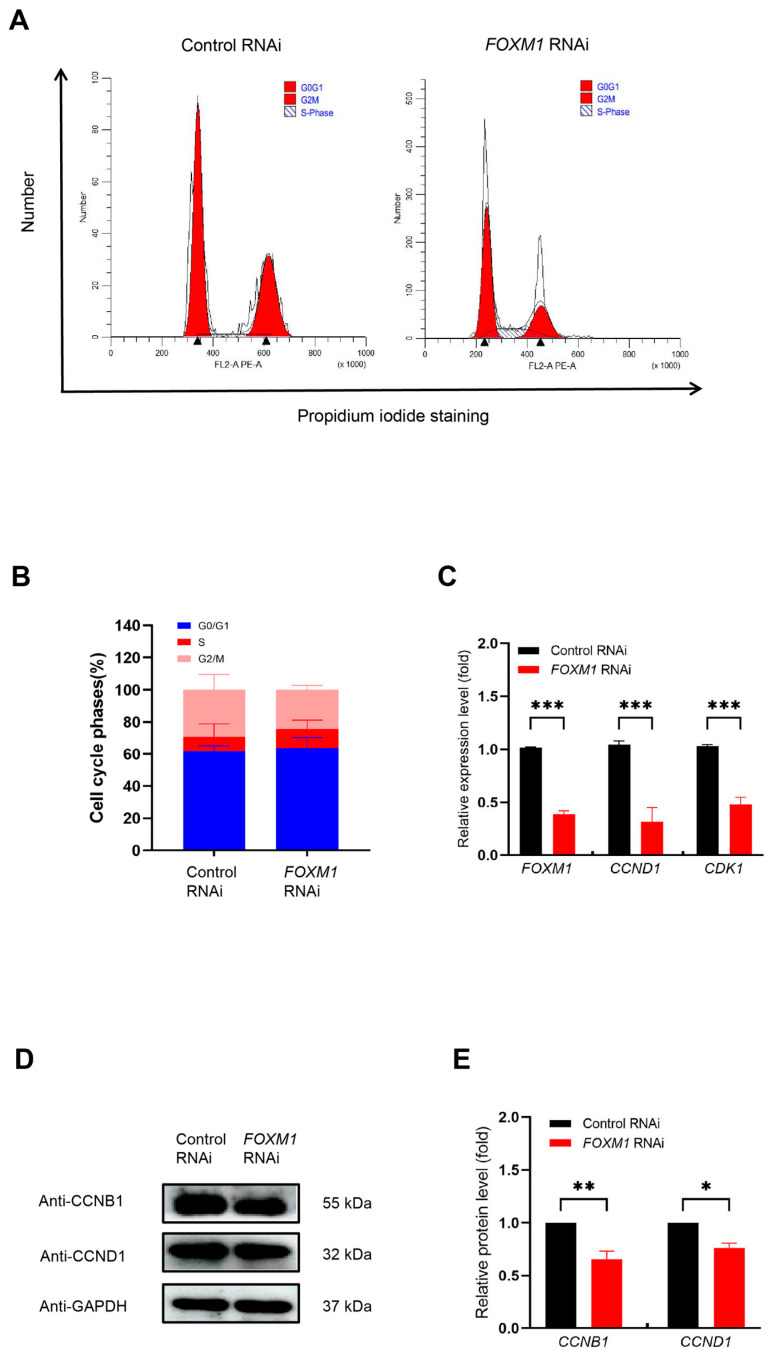
Knockdown of *FOXM1* affects cell cycle progression. (**A**) Cell cycle progression was examined by flow cytometry. (**B**) The relative expression levels at different stages of the cell cycle were displayed using stacked plots. (**C**) RT-qPCR was used to detect the relative expression levels of *CCND1* and *CDK1* genes. (**D**) The relative expression of cell cycle proteins was determined by Western blotting. (**E**) Quantification of cell cycle proteins. * *p* < 0.05, ** *p* < 0.01, and *** *p* < 0.001.

We further investigated the effects of *FOXM1* knockdown on cell cycle progression in WJ-MSCs. Initially, we examined cell cycle distribution using flow cytometry. We found that *FOXM1* RNAi led to an increase in the number of cells in the G0/G1 phase, along with a decrease in the number of cells in the S and G2/M phases (Figure 5A,B). These results suggest that FOXM1 has an important regulatory role in the cell cycle.

To investigate the molecular mechanisms by which FOXM1 impacts the cell cycle, we utilized RT-qPCR to measure the expression levels of key regulatory genes (*CCND1* and *CDK1*). Our results demonstrated that *FOXM1* RNAi significantly reduced *CCND1* and *CDK1* mRNA expression to less than 50% (Figure 5C, *** *p* < 0.001). These findings, aligning with the RNA-seq data, reinforced that FOXM1 may influence cell cycle progression through the regulation of these genes. Additionally, we validated the protein expression levels of the key cell cycle proteins CCNB1 and CCND1 via Western blot. Our results showed that *FOXM1* RNAi significantly diminished CCNB1 and CCND1 protein levels (Figure 5D,E, ** *p* < 0.01). These results are consistent with the changes in gene expression and further emphasize the critical role of FOXM1 in cell cycle regulation.

In summary, our findings establish that FOXM1 sustains WJ-MSC proliferation by directly promoting cell cycle progression; its knockdown dysregulates cell cycle genes, inducing a G0/G1 arrest and halting proliferation.

### 3.6. FOXM1 Knockdown Promotes Apoptosis in WJ-MSCs

To validate the RNA-seq findings and further investigate FOXM1’s regulatory role, we confirmed the expression of key differentially expressed genes using RT-qPCR. The RNA-seq data had initially indicated that *FOXM1* knockdown significantly altered the transcriptome, with GO and KEGG analyses linking these changes to critical processes such as cell proliferation, apoptosis, inflammatory response, and stem cell maintenance (Figure 3).

**Figure 6:**
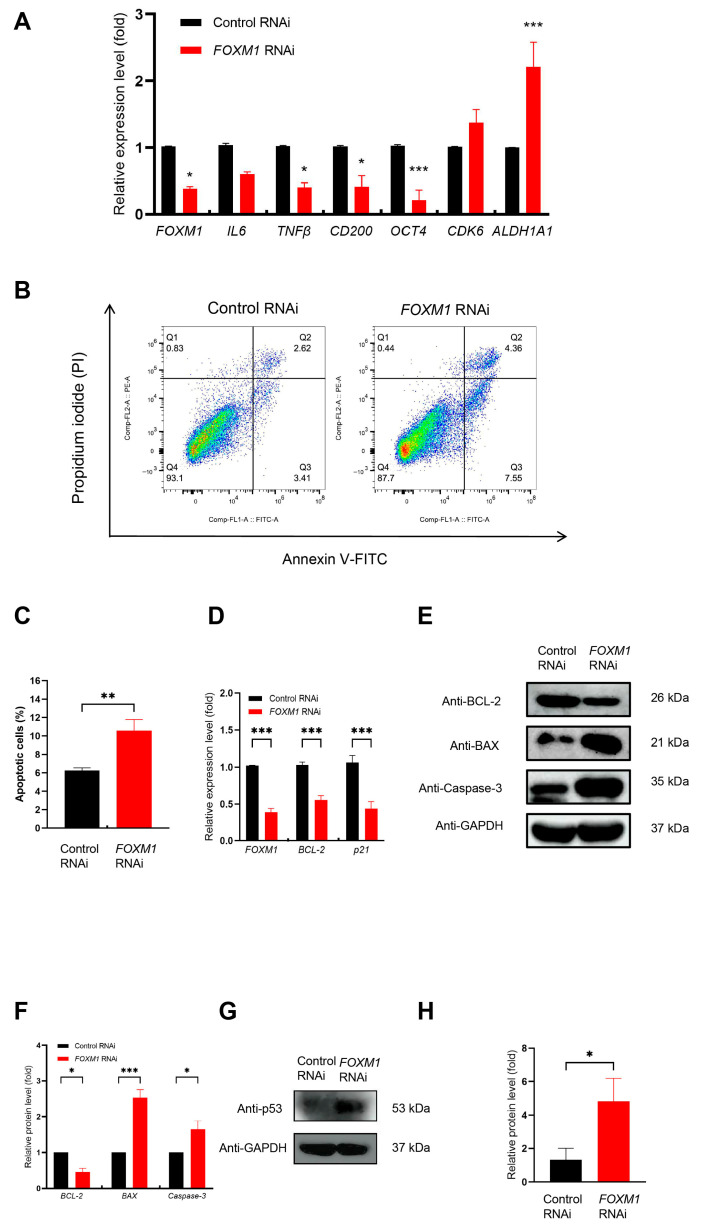
*FOXM1* knockdown affects gene expression in multiple biological processes and promotes cell apoptosis. (**A**) Relative gene expression changes in *IL6*, *TNFβ*, *CD200*, *OCT4*, *CDK6*, and *ALDH1A1* upon FOXM1 RNAi. (**B**) Apoptosis caused by *FOXM1* knockdown was assessed by flow cytometry using Annexin V-FITC/PI staining. (**C**) After *FOXM1* knockdown, apoptotic cells from three independent experiments were analyzed by FlowJo, and the results were shown in a bar chart. (**D**) RT-qPCR was used to detect the changes in *BCL-2* and *p21* gene expression. (**E**) The relative expression of apoptotic proteins was detected by Western blotting. (**F**) Quantification of apoptotic proteins. (**G**). The relative expression of p53 protein was detected by Western blotting. (**H**) Quantification of the p53 protein. * *p* < 0.05, ** *p* < 0.01, and *** *p* < 0.001.

To validate the reliability of the RNA-seq results, we selected representative genes from the significantly enriched key pathways for RT-qPCR analysis. These genes included *IL6*, *TNFβ*, *CD200*, *OCT4*, *CDK6*, and *ALDH1A1* (Figure 6A). We observed a significant downregulation of genes critical for inflammation, immune modulation (*IL6*, *TNFβ*, *CD200*), and stem cell identity (*OCT4*, *ALDH1A1*), alongside dysregulation of the cell cycle gene *CDK6*. The pronounced decrease in *CD200* provides a clear validation of the sequencing data. Collectively, these results verify that *FOXM1* knockdown disrupts core cellular functions, ultimately leading to the loss of stem cell characteristics in WJ-MSCs.

To determine whether the observed G0/G1 phase cell cycle arrest and inhibited proliferation (Figure 5) upon *FOXM1* knockdown were coupled with an apoptotic response, we investigated its effects on apoptosis and associated mechanisms. The apoptosis pathway was prominently enriched in our KEGG analysis and displayed a global upregulation pattern (Figure S1B). Flow cytometry analysis revealed a significantly higher apoptosis rate in FOXM1-deficient cells compared to controls, with a notable increase of 5.88% (Figure 6B,C). This was supported by a significant reduction in the expression levels of BCL-2 and p21 (Figure 6D). Critically, the analysis of protein levels provided additional evidence for pathway activation. These results together indicate that the reduction in FOXM1 may promote cell death. Western blot analysis confirmed these findings, demonstrating a decrease in the anti-apoptotic protein BCL-2 and a concomitant increase in the pro-apoptotic proteins BAX and Caspase-3 (Figure 6E). Quantitative analysis of the protein levels confirmed these changes were statistically significant (Figure 6F).

To clarify the upstream mechanism by which FOXM1 deficiency triggers this apoptotic cascade, we studied the change in p53, a main regulatory factor of the intrinsic apoptosis pathway. Compared with the control group, *FOXM1* knockdown significantly led to an upregulation of p53 protein expression (Figure 6G,H). This finding indicated that p53 accumulation may be associated with FOXM1 inhibition in WJ-MSCs. Collectively, these results demonstrated that FOXM1 may be a critical regulator of apoptosis in WJ-MSCs.

## 4. Discussion

Previous research has shown that FOXM1 plays a significant role in the proliferation [33], differentiation, and survival of various cell types [34]. However, its specific functions and molecular mechanisms in WJ-MSCs remain unclear. In this study, our data suggests that FOXM1 acts as an important transcription factor for maintaining the homeostasis of WJ-MSCs, with its knockdown impacting multiple functions of these cells. We systematically explored its potential roles in proliferation, migration, cell cycle progression, apoptosis, and stem cell pluripotency in vitro.

FOXM1 appears to support the colony-forming ability, proliferation, and migration of WJ-MSCs. Our data indicate that *FOXM1* knockdown significantly impairs the colony-forming potential of WJ-MSCs, leading to reduced cell proliferation and migration. Consistent with the downregulation of CCND1 and G1 phase cell cycle arrest, CCK-8 assays demonstrated that *FOXM1* knockdown significantly diminished WJ-MSC proliferation. These findings reinforce the notion that FOXM1 may be indispensable for the proliferation of WJ-MSCs. Numerous studies have shown that FOXM1 promotes cell proliferation [35] and migration [36], and support the notion that FOXM1 contributes to the proliferative capacity of WJ-MSCs.

Additionally, FOXM1 impairs the stem cell characteristics and cell cycle regulation of WJ-MSCs. OCT4, NANOG, and SOX2 are well-known core transcription factors that sustain the self-renewal and pluripotency of human pluripotent stem cells [37,38]. Our results showed that *FOXM1* RNAi decreased the expression of pluripotency-related genes, including OCT4, SOX2, and NANOG. This is consistent with a previous study which has identified FOXM1 as a key target upstream of the pluripotency regulatory network, which directly binds to the *OCT4* promoter to maintain the expression of pluripotency markers [39]. However, since OCT4, SOX2, and NANOG are expressed at much lower levels in WJ-MSCs, whether this direct regulatory mechanism operates in WJ-MSCs still remains to be experimentally tested. Additionally, FOXM1 acts as a transcriptional activator in cell cycle regulation, playing a significant role in the G1/S and G2/M transitions [40,41]. We hypothesize that the cell cycle arrest and proliferation defects caused by *FOXM1* knockdown are a synergistic process driven by both direct and indirect mechanisms. On the direct level, *FOXM1* knockdown may lead to the inactivation of its downstream targets, such as *CCNB1* and *CDK1*, which directly impede cell cycle progression. On the indirect level, *FOXM1* knockdown results in decreased expression of OCT4, whose deficiency has been shown to induce cell cycle arrest and impair proliferation [42]. Thus, the concurrent disruption of these key cellular processes upon *FOXM1* knockdown demonstrates its central role in sustaining WJ-MSC homeostasis. Future studies employing promoter-binding assays and other mechanistic experiments are necessary to definitively establish if FOXM1 directly regulates those genes in WJ-MSCs.

Moreover, we also found that FOXM1 knockdown relieved the inhibition of pro-apoptotic factors such as BAX, while the subsequent upregulation of Caspase-3 marked the initiation of the apoptotic execution phase. Critically, our data show that this process is likely mediated by the tumor suppressor p53, whose protein level is significantly upregulated upon *FOXM1* knockdown. As a master transcriptional regulator of apoptosis, p53 can directly transcribe and activate the apoptotic gene *BAX* and inhibit the expression of anti-apoptotic gene *BCL-2* [43,44], thus leading to an increase in the *BAX/BCL-2* ratio and initiating the apoptotic pathway. Therefore, the accumulation of p53 resulting from *FOXM1* knockdown provides a logical explanation for the concurrent upregulation of *BAX* and downregulation of *BCL-2.* The resulting shift in the *BAX/BCL-2* ratio ultimately leads to the activation of Caspase-3 and triggers programmed cell death. Taken together, our data supports a model in which FOXM1 acts as a negative regulator of p53 in WJ-MSCs, which in turn orchestrates the apoptotic pathway by modulating the expression of BAX and BCL-2. Collectively, our data establishes a coherent link between *FOXM1* knockdown and the activation of intrinsic apoptosis in WJ-MSCs. This conclusion is supported by a definitive chain of evidence spanning three levels: initial pathway enrichment prediction, subsequent validation of key regulatory proteins, and ultimate confirmation of the apoptotic phenotype. This finding aligns with previous research establishing FOXM1 as a critical survival factor in cancer cells [25,45], where its inhibition can induce apoptosis [46]. However, the exact interplay between these processes and whether they form a feedback loop remains to be experimentally validated.

In addition to its coordinated regulation of the cell cycle, proliferation, apoptosis, and pluripotency, we have found that FOXM1 influences the biological behavior of WJ-MSCs by regulating several key genes. These genes have been found to be involved in processes such as promoting angiogenesis and immunomodulation in other contexts, such as *IL6*, *TNFβ, CD200, OCT4, CDK6*, and *ALDH1A1*. Therefore, maintaining the expression level of these genes may be crucial to the therapeutic effect of WJ-MSCs. Although this implies that FOXM1 may have a broader potential role in regulating the secretion and functional characteristics of WJ-MSCs, the direct experimental verification of its specific functional results still needs to be explored in future research. In general, our findings provide new mechanistic insights into how FOXM1 governs the therapeutic potential of WJ-MSCs.

This study systematically reveals the multifaceted functions of FOXM1 in WJ-MSCs through gene regulatory mechanisms, providing a vital theoretical basis for optimizing stem cell expansion therapies. Additionally, this research offers new perspectives for the fields of stem cell biology and regenerative medicine. However, this study has several limitations. First, the limitations of this study include its primary focus on in vitro experiments; the specific mechanisms of FOXM1 in vivo still require further investigation. Second, all experiments use single commercial source cell lines. Although this will avoid the complex interference caused by heterogeneity between donors, it may not be universal. Third, while we observed changes in gene expression following *FOXM1* knockdown, Chromatin immunoprecipitation using FOXM1 antibody to validate the direct binding of FOXM1 to the genomic sites of its downstream genes is required to confirm the direct transcriptional regulatory role of FOXM1. Fourth, the functional consequences of altered genes (*IL6*, *CD200*, *ALDH1A1*) were not experimentally validated in therapeutic models. Fifth, future studies can include TUNEL staining or Caspase activity assays which are important to confirm and quantify the apoptosis induced by *FOXM1* knockdown. It is also important to verify these findings in vivo.

## 5. Conclusions

In summary, our study establishes FOXM1 as a master regulator of WJ-MSC homeostasis. We demonstrate that it sustains proliferation by promoting cell cycle progression and concurrently suppressing apoptosis and stress signals. Consequently, FOXM1 depletion critically impairs cellular expansion, underscoring its vital, non-redundant role in maintaining the integrity and function of the WJ-MSC population.

## Data Availability

The original contributions presented in this study were included in the article/Supplementary Materials. Further inquiries can be directed to the corresponding author.

## Supplementary Materials

The following supporting information can be downloaded at: https://www.mdpi.com/article/10.3390/genes16121517/s1, Figure S1: Key Pathways Altered by FOXM1 Knockdown. Table S1: Primer sequences of target genes in qPCR. Table S2. Primary and secondary antibodies being used.

## Abbreviations

The following abbreviations are used in this manuscript.

WJ-MSC	Wharton’s jelly mesenchymal stem cell
MSC	Mesenchymal stem cells
FOXM1	Forkhead box protein M1
RT-qPCR	Real-Time Quantitative PCR
PI	Propidium iodide
KEGG	Kyoto Encyclopedia of Genes and Genomes
GO	Gene Ontology
