# Supplementary material for: FOXM1 Maintains Homeostasis and Self-Renewal in Wharton’s Jelly Mesenchymal Stem Cells
Source: Genes (Basel). 2025 Dec 18;16(12):1517. doi: 10.3390/genes16121517 (PMC12732837; doi:10.3390/genes16121517)
Supplement: Supplementary file 1 [file genes-16-01517-s001.zip › genes-4028179-supplementary.pdf]

Supplementary Materials for

FOXM1 Maintains Homeostasis and Self-Renewal in Wharton's Jelly Mesenchymal Stem Cells

Supplementary Figure S1

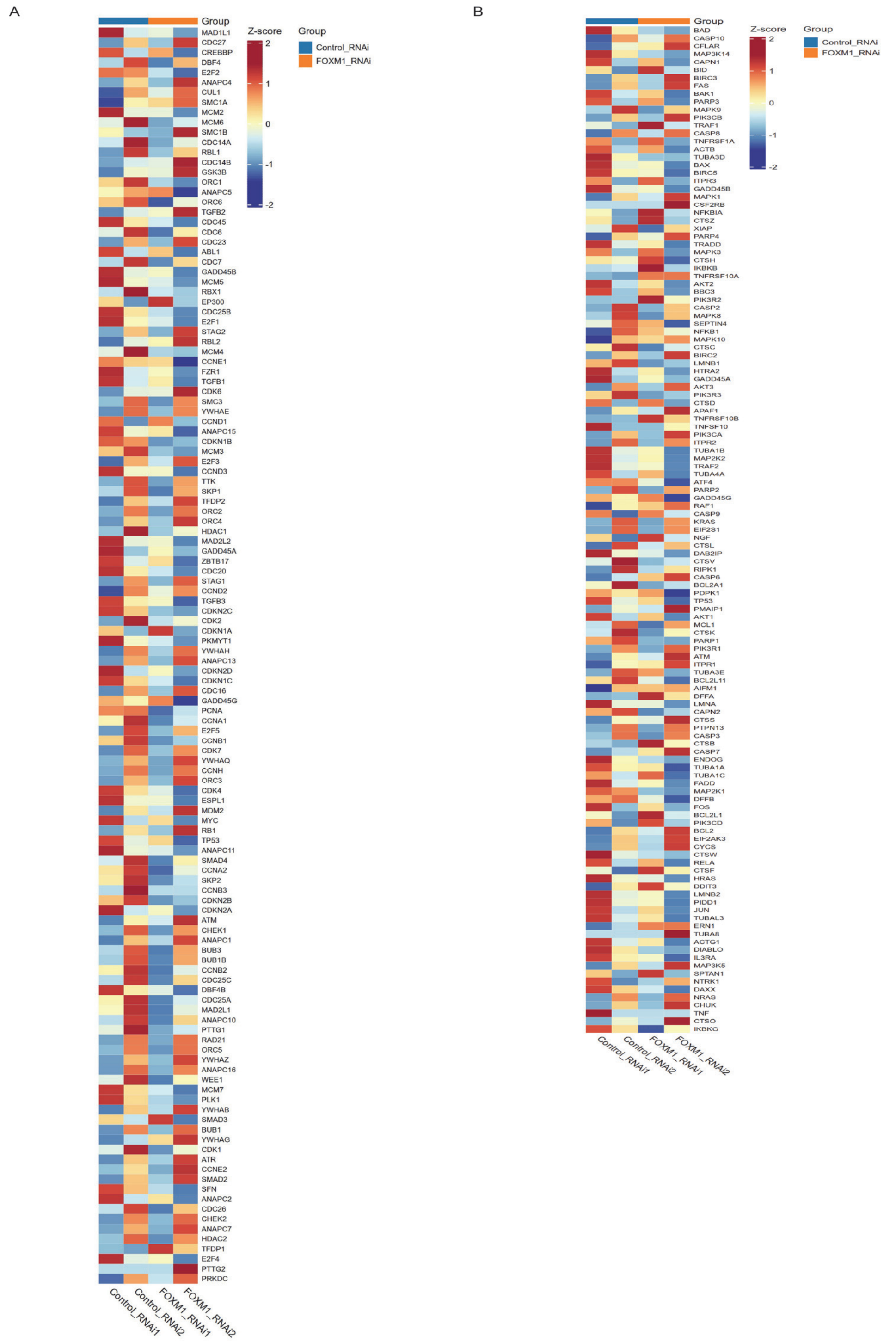

Figure S1. Key Pathways Altered by *FOXM1* Knockdown. (A) Heatmap displaying the expression of all genes within the Cell Cycle pathway. (B) Heatmap displaying the expression of all genes within the Apoptosis pathway.

Supplementary Table S1: Primer sequences of target genes in qPCR.

Gene	Primer Sequences (5′-3′)
GAPDH（Human）	F:GAAGGTGAAGGTCGGAGTCAACGGA
	R:AATTTGCCATGGGTGGAATCATATTGGAA
FOXM1（Human）	F:AGCGACAGGTTAAGGTTGAG
	R:GATAGGCACCAGGTATGAGC
IL6（Human）	F:AGACAGCCACTCACCTCTTCAG
	R:TTCTGCCAGTGCCTCTTTGCTG
TNFβ（Human）	F:AGGGCTACCATGCCAACTTC
	R:GACACAGAGATCCGCAGTCC
CD200（Human）	F:GAAGGTCTCAGGAACAGCTTGC
	R:GCAGTCGCAGAGCAAGTGATGT
ALDH1A1（Human）	F:GGAATACCGTGGTTGTCAAGCC
	R:CCAGGGACAATGTTTACCACGC
Ki-67（Human）	F:GAAGCCCATGAAGACCTCCC
	R:CGCTCTCCTCTGCCACCTTA
CDK6（Human）	F:CAGCCTGCAGGAAGCATGAG
	R:CAAAGTAAAGAACCGAGATG
OCT4（Human）	F:TTGGGCTAGAGAAGGATGTGGTT
	R:GGAAAAGGGACTGAGTAGAGTGTGG
CCND1（Human）	F:GCATGTTCGTGGCCTCTAAGA
	R:CGGTGTAGATGCACAGCTTCTC
CDK1（Human）	F:CAGAAGTGGAATCTTTACAGGACTATAAGA
	R:GATCATAGATTAACATTTTCGAGAGCAA
BCL-2（Human）	F:CTGGCATCTTCTCCTTCCAG
	R:GACGGTAGCGACGAGAGAAG
p21（Human）	F:CGGTGGAACTTTGACTTCGT
	R:CAGGGCAGAGGAAGTACTGG

Supplementary Table S2. Primary and secondary antibodies being used.

Protein specificity	Source	Identifier	Dilution Ratio
Anti-GAPDH	Cell Signaling Technology	51332S	1:1000
Anti-FOXM1	Santa Cruz	sc-271746	1:500
Anti-OCT4	Santa Cruz	sc-5279	1:500
Anti-NANOG	Santa Cruz	sc-134218	1:500
Anti-SOX2	Santa Cruz	sc-365823	1:1000
Anti-CCNB1	ZenBio	340296	1:1000
Anti-CCND1	Santa Cruz	sc-8396	1:1000
Anti-BCL-2	Santa Cruz	sc-7382	1:1000
Anti-BAX	Santa Cruz	sc-7480	1:1000
Anti-Caspase-3	Santa Cruz	sc-7272	1:1000
Anti-p53	Cell Signaling Technology	9282	1:1000
Anti-Mouse IgG- HRP	Santa Cruz	sc-2005	1:5000
Anti-Rabbit IgG-HRP	Santa Cruz	sc-2004	1:5000
